# Challenges in the Management of Chronic Urticaria

**DOI:** 10.1097/1939-4551-4-S3-S28

**Published:** 2011-03-15

**Authors:** Todor A Popov

**Affiliations:** 1 Clinic of Allergy & Asthma, Medical University, Sofia, Bulgaria

**Keywords:** chronic idiopathic urticaria, histamine, nonsedating antihistamines, levocetirizine, desloratadine

## Abstract

The term "chronic idiopathic urticaria" denotes a spectrum of conditions with different poorly understood pathogenetic mechanisms in which the release of histamine plays a role. Nonsedating second-generation H_1 _antihistamines are postulated to be the first line of treatment of chronic idiopathic urticaria by national and international guidelines, but as control is not always achievable with the usually recommended doses, first-generation sedating antihistamines like hydroxyzine and diphenhydramine at high daily doses (200 mg) have been proposed as an alternative before resorting to treatment with systemic corticosteroids and other potentially hazardous agents. Our long time experience and recent research give us grounds to believe that increasing the doses of nonsedating H_1 _antihistamines up to fourfold improves significantly the chances of successful treatment. Our data suggest that the urticaria-associated discomfort is relieved by higher than conventional doses of levoce-tirizine and desloratadine in about 75% of the patients and that sedation/somnolence does not seem to be a major deterrent. The dose increase also improves the urticaria-specific quality of life. Contrary to the belief that individual patients may benefit from one antihistamine or another, we demonstrate that the drug with better ability to suppress the histamine skin effects in experiments in healthy volunteers (levocetirizine) is also superior in improving the different aspects of control of chronic urticaria (subjective and objective symptoms, quality of life) and that increasing its dose of up to fourfold may even paradoxically reduce the sense of sedation/somnolence in parallel with the relief of urticaria discomfort.

## Defining the Topic

**Figure 1:**
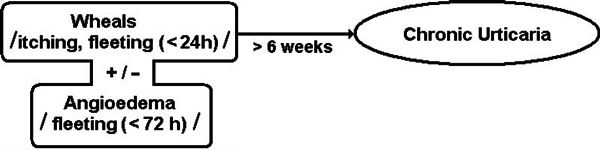
**Graphical definition of chronic urticaria**.

Awide spectrum of different conditions share a seemingly common clinical feature: appearance of urticarial lesions. The latter are described as red, raised, itchy wheals ("nettle rash," "hives") resulting from vasodilatation, increased blood flow, and increased vascular permeability[1]. They have fleeting character and disappear without a trace in less than 24 hours. In a significant proportion of the cases they can be accompanied by swelling of the deeper cutaneous and subcutaneous tissues referred to as angioedema, tending to regress substantially slower (up to 72 hours). When the wheals with or without angioedema recur for longer than 6 weeks, they fall within the definition of chronic urticaria (Figure 1).

Chronic urticaria (CU) is a relatively common condition, which has a profound effect on the quality of life of those suffering[2]. The need to establish an optimal approach to this class of disorders resulted in working out of national and international guidelines for its diagnosis, classification, pathogenetic mechanisms, and management[3,4]. As opposed to other types of urticaria like acute urticaria, physical urticaria, and urticarial vasculitis that may have identifiable causes and triggers, the nature of chronic spontaneous urticaria remains elusive, which is reflected in the commonly accepted term chronic idiopathic urticaria (CIU). In about 45% of the cases of CIU, signs of autoimmunity can be detected by means of skin tests to autologous serum or plasma, or by detection of autoantibodies to IgE or the high-affinity IgE receptor (Fc*ε*RI) on basophils and mast cells[4-6]. Another clue toward autoimmunity is evidence of an increased prevalence of antithyroid autoantibodies among CU patients[7]. It appears, though, that identifiable autoimmune mechanisms do not alter the subsequent treatment approach. The latest EAACI/GA(2)LEN/EDF/WOA management guideline grades the strength and quality of the experimental evidence when formulating recommendations[8]. The starting point for pharmacological treatment is the prescription of nonsedating H_1 _antihistamines (nsAH) (table 1, adapted from Table in reference 8).

**Table 1:** EAACI/GA(2)LEN/EDF/WOA Algorithm for Pharmacological Treatment of CIU[8]

**nsAH**
↓ If symptoms persist after 2 weeks:
nsAH updosing (up to 4 times)
↓ If symptoms persist after 1-4 weeks:
Add leukotriene antagonist or change nsAH
Exacerbation: systemic steroids (for 3-7 days)
↓ If symptoms persist after 1-4 weeks:
Add cyclosporine A, H_2 _antihistamine,^a ^dapsone, omalizumab
Exacerbation: systemic steroids (for 3-7 days)

^a^In the United States, adding H2-receptor antagonist to the H1 antihistamine comes as a second step in the CU management algorithm.

When analyzing the state-of-the-art of urticaria management, the guidelines have recognized deficits in the supporting evidence. In a recent overview of his extensive experience in urticaria treatment, Allen Kaplan, a leading expert in the field of urticaria, lists a number of misconceptions among the medical community and the lay public related to urticaria management and pinpoints controversies relating to the use of AH, which need to be addressed by specifically designed clinical studies[9]. The present paper is focusing exclusively on the treatment of chronic urticaria with nsAH, as these drugs are suggested as the first line of treatment, but unresolved issues still remain because of the relative paucity of reliable evidence[8].

## The Role of Histamine in CIU

Histamine is a low molecular weight amine synthesized from L-histidine by the enzyme histidine decarboxylase and plays a vital role in the regulation of many important functions of the organism related to circadian influences, adaptation to the environment, and stress. Conversely, histamine is intimately implicated in the pathogenesis of allergic and other diseases, which develop as a defective systemic trait and may have different organ expressions. Histamine exerts its multiple effects by coupling to receptors expressed on the membrane of cells in different tissues. Four different receptors have been identified and sequenced so far denoted sequentially as H_1 _to H_4_[10]. The histamine effects in the skin associated with urticaria, particularly the wheals and the itch, are mediated through H_1 _receptors located on nerves and endothelial cells[11]. A role in urticaria has been proposed also for the H_4 _receptors in the generation of pruritus and eosinophil chemotaxis[12]. The histamine receptors exhibit stereochemical differences, which can be recognized by different chemical compounds, referred to as specific receptor agonists. A big proportion of these invert the original effects of histamine by rendering the receptor-G-protein complex inactive, which categorizes them as "inverse" agonists[13]. However, as they abolish the effects of histamine, they are popularly known as histamine receptor antagonists or antihistamines.

The mechanisms at play in patients with chronic urticaria are much more complex than just release of histamine from basophils and mast cells triggered by a variety of pathways[6]. Involvement of other mediators like leukotrienes, prostaglandins, kinins, anaphylatoxins, and chemokines is also occurring, shaping individual patterns in each particular case and determining the success of treatment[14]. A cellular infiltrate similar to a late-phase IgE-mediated allergic reaction with ensuing therapeutic implications has been extensively reviewed[15]. Still, in most cases histamine has a leading role in the pathogenesis of chronic urticaria, which is the reason for H_1 _AH to be proposed as first-line and mainstay treatment (Table 1)[8].

## Picking the Best Antihistamine for the Treatment of CIU

Within a period of 40 years since 1937, when Bovet and Staub described the first pharmaceutical formulation to counter the effects of histamine in a guinea pig, about 40 AH had been introduced for the treatment of nasal allergic and skin symptoms. They all had a short plasma half-life, were not very H_1_-receptor specific, caused significant central nervous system (CNS) adverse effects, and became subsequently known as first-generation H_1 _AH[16]. Some of them like hydroxyzine and diphenhydramine had proven to be of particular benefit in urticaria and are still considered as possible choices in cases resistant to treatment[17]. A significant step forward was the synthesis of more complex compounds with improved pharmacokinetic and pharmacodynamic profiles: they hardly penetrated the blood-brain barrier, which makes them relatively free of the most bothersome adverse CNS effects (drowsiness, lassitude, dizziness, and incoordination), possessed higher specificity for the H_1 _receptors, and had longer half-life, allowing a once daily dosing regimen[18]. They were referred to as second-generation H_1_-receptor antagonists, and whereas 2 of the earlier compounds (terfenedine and astemisol) were abandoned because of serious cardiac side effects, the "newest arrivals" (fexofenadine, desloratadine, and levocetirizine) took up the lead in prescription practices as "modern" second-generation drugs.

Ideally, an AH drug should bind to a maximal number of H_1 _receptors to ensure the best effect. Determinants for this to happen are the applied dose and the pharmoacokinetic properties of the separate formulations: bioavailability, plasma half-life, volume of distribution, and receptor affinity. Following this rationale, the second-generation nsAH should have a clear cut advantage over the first-generation ones. This is not, however, the case in everyday practice where nonexperts treat urticaria and prescribe first-generation drugs contrary to guidelines[19]. At least in part this is due to the belief that sedation may be helpful in soothing the itch in urticaria patients.

The debate about the use of first-generation H_1 _AH is still alive because of some other arguments prompted by empirical experience. Thus, Allen Kaplan is favoring high doses of hydroxyzine and diphenhydramine if doubling the doses of second-generation nsAH does not achieve satisfactory control of symptoms[9,15,17]. In the absence of appropriate studies to provide conclusive evidence, he backs up his assertions with compelling reasoning:

• The relatively lower specificity of the first-generation AH for the H_1 _receptors may prove an advantage as they may exert some additional beneficial effects through other mechanisms: antimuscarinic, anti-*α*-adrenergic, antiserotonin effects, and activity against H_4 _receptors.

• Sedation, somnolence, and performance impairment with H_1 _AH are considered a limitation to their use. However, unlike other conditions where histamine plays a role, these features in urticaia patients are intimately associated with the nature of the disease, as sleep deprivation because of nighttime itch can be the real cause underlying them. Thus, sedating AH may turn out to have a paradoxal positive effect on the state of alertness.

• The sedative effects on initial intake are short-lived and gradually wear off by day 4 of treatment with first- generation AH.

• The alternative to sedation would be the prescription of corticosteroids and other more hazardous and expensive drugs like cyclosporine, methotrexate, sulfasalazine, intravenous gamma globulin, dapsone, hydroxychloroquine, colchicine, and cytoxan, with the ensuing deleterious risks in the long run.

This sound logic is backed up by decades of first-generation AH prescriptions, indicating that really high doses of some of the older drugs can be used to saturate all skin H_1 _receptors. Thus, Allen Kaplan advocates that up to 4 × 50 mg of hydroxyzine and diphenhydramine should be applied to increase the chances of success if doubling the standard dose of nsAH fails. This leads us to a total daily dose of 200 mg of these first-generations drugs, which is way too high compared with the 20-mg daily dose of cetirizine, if a speculative approximation of 10 mg of cetirizine corresponding to 30 mg of hydroxyzine (used in comparative studies) is assumed. The question emerges whether increasing the dose of nsAH high enough would prove helpful in controlling the urticaria symptoms. Studies with fexofenadine failed to show that increasing the dose from 60 to 240 mg makes a meaningful difference[20,21]. However, a twofold increase of the standard dose of cetirizine was found effective in patients with cholinergic urticaria,[22] a threefold increase of the cetirizine dose brought about some benefit in a small proportion of patients with chronic unremitting urticaria,[23] and a fourfold increase of the recommended dose of desloratadine was proven effective in acquired cold urticaria[24].

**Figure 2:**
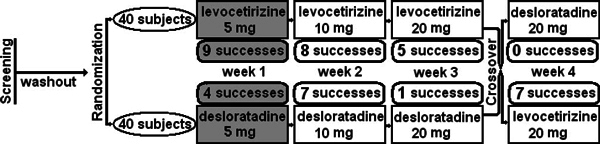
**Results of the study of higher than conventional doses of levocetirizine and desloratadine in difficult-to-treat urticaria (adapted from Staevska et al**[25]**)**. The darkened boxes represent the successes at conventional doses for both drugs and the light boxes represent the additional value of dose increase.

Led by the plausible statement of Allen Kaplan that defining the level of refractoriness to antihistamines is the key to effective therapy, we carried out a study to check if a dose increase of the modern second-generation H_1 _receptor antagonists desloratadine and levocetirizine of up to fourfold could increase the success rate of treatment of chronic urticaria and improve the health-related quality of life without compromising safety[25]. We recruited 80 patients with difficult-to-treat chronic urticaria defined on the basis of referral to our tertiary specialist clinic after previous treatment failures, and, after documentation of their subjective symptoms and quantification of the objective findings, randomized them to receive in a double-blind crossover fashion increasing doses of either levocetirizine (40 patients) or desloratadine (40 patients) at 1-week intervals. The primary outcome was the number of patients becoming symptom-free (no subjective symptoms and no urticarial lesions) at the different dosing steps; the secondary outcomes included assessment on 100-mm visual analog scales of the discomfort because of urticaria and the level of sedation/somnolence, and the quality of life evaluated by means of Chronic Urticaria Quality of Life Questionnaire[26]. The results demonstrated that the number of patients rated as having treatment success significantly increased (more than doublefold) at doses higher than the conventional 5-mg dose (Figure 2).

A common belief is that patients might respond to one or another AH depending on their specific phenotype, rather than on the pharmacological characteristics of the different formulations. This type of reasoning prompts switching from one drug to another and also simultaneous intake of different AH. In a preceding study we compared the ability of increasing doses of levocetirizine and desloratadine to suppress the effects of intradermal histamine in healthy men[27]. The results indicated that levocetirizine is significantly more effective than desloratadine in inhibiting the wheal and flare responses to histamine in human skin in vivo, with 1.25 mg of levocetirizine being more effective than 10 mg of desloratadine. This superior effectiveness of levocetirizine in the skin was confirmed in our above-described clinical study in chronic urticaria patients, in which 7 patients who failed to respond to 20 mg of desloratadine became symptom-free on levocetirizine, whereas none of the fewer therapeutic failures at 20 mg of levocetirizine benefited from the switchover to 20 mg of desloratadine. The higher efficacy of levocetirizine than that of desloratadine in CIU was also confirmed in a multicenter study of 886 patients using only the standard 5-mg dosage[28].

The relationship between subjective perceptions like discomfort because of urticaria, sedation/somnolence, and disease-specific quality of life is rather complex. On the basis of the relief of the discomfort because of urticaria, we could differentiate "low-dose responders," approximately 10% of the patients who responded to the standard dose of 5 mg, "nonresponders," about 15% who did not experience any improvement even at the highest 20-mg dose, and "high-dose responders," ranging in the gray zone between those 2 extremes making up 75% of the patients[25]. In the majority of patients somnolence (measured on a 100-mm visual-analog scale at baseline and after each treatment step) did not change or decreased in the course of the incremental treatment. This finding was better expressed with levocetirizine, where also a significant correlation shaped up between the positive change in the perception of urticaria discomfort and the reduction of somnolence. The analysis of the quality of life questionnaires revealed improvement with the increasing doses of both drugs, still better outlined with levocetirizine.

## Conclusions

The quoted study and the experience we have acquired during the past years in the treatment of chronic urticaria give us ground to believe that high enough doses of nsAH give the best chance of achieving control of the urticaria symptoms before resorting to other potentially more hazardous approaches. The higher doses of these drugs may also be associated with additional anti-inflammatory effects[29] that may be relevant for many cases of chronic urticaria. Present and future formulations with the highest affinity for the skin should be given preference in more severe cases of chronic urticaria. The controversy about the use of first-generation versus second-generation H_1 _AH in chronic urticaria could be given an ultimate answer only if specifically designed comparative studies are carried out.
